# Perioperative effects of caudal block on pediatric patients in laparoscopic upper urinary tract surgery: a randomized controlled trial

**DOI:** 10.1186/s12887-019-1812-0

**Published:** 2019-11-11

**Authors:** Bingdong Tao, Kun Liu, Dandan Wang, Mengmeng Ding, Ni Yang, Ping Zhao

**Affiliations:** 1 Department of Anesthesiology, Shengjing Hospital, China Medical University, 36 Sanhao Street Heping District, Shenyang, 110004 Liaoning Province China; 2 Department of Pediatrics, PICU, Shengjing Hospital, China Medical University, Shenyang, China

**Keywords:** Caudal block, Pediatric surgery, Upper urinary tract surgery, Laparoscopic surgery, Ropivacaine

## Abstract

**Background:**

While caudal block has been widely used during pediatric lower limbs and lower abdominal surgeries, few studies to date have evaluated the perioperative effects of caudal block on pediatric patients in laparoscopic upper urinary tract surgery.

**Methods:**

Ninety-six pediatric patients, aged 6 months to 7 years, ASA grade I-II, scheduled to undergo laparoscopic upper urinary tract surgery, were randomized to a non-block group (no caudal block performed), an ROP1.0 group (patients received 1.0 mL/kg of 0.15% ropivacaine) and an ROP1.3 group (patients received 1.3 mL/kg of 0.15% ropivacaine). The primary outcome variable was perioperative fentanyl use. The secondary outcome variables were pain score, hemodynamic fluctuation, the number of patients needing rescue fentanyl and side effects.

**Results:**

Caudal block with 1.3 mL/kg of 0.15% ropivacaine significantly decreased perioperative fentanyl usage (ROP 1.3 vs. non-caudal block, *P* < 0.01; ROP 1.3 vs. ROP 1.0, *P* < 0.05). Moreover, patients in the ROP1.3 group, compared to those without, displayed more stable hemodynamics, lower pain score in the PACU and 8 h after operation, less demand for rescue fentanyl, shorter time of PACU stay.

**Conclusions:**

Caudal block with 1.3 mL/kg of 0.15% ropivacaine reduced perioperative fentanyl use during laparoscopic upper urinary tract surgery on pediatric patients and produced good postoperative analgesia when compared with no caudal block and caudal block with 1.0 mL/kg of 0.15% ropivacaine.

**Trial registration:**

Clinical trial number: ChiCTR1800015549, chictr.org.cn.

## Background

It is well known that pediatric laparoscopic surgery has many advantages, such as minimal injury, invisible scar, fast recovery, and shortened hospital stay [1, 2]. However patients will still suffer from medium to severe postoperative pain, and pneumoperitoneum in laparoscopy often causes instable hemodynamics [3–5]. In order to control postoperative pain and maintain stable hemodynamics, opioids are often used during laparoscopic surgery [6, 7], but increased opioids usage is correlated to high occurrence of postoperative nausea and vomiting, respiratory depression, prolonged hospital stay, and so on [8–10].

Caudal block, a well-established technique in pediatric surgeries such as lower limb surgery and lower abdominal surgery, can produce good analgesic effects and maintain stable hemodynamics [11–13]. Several studies show that caudal block also produces good postoperative analgesic effects after pediatric laparoscopic surgery [14, 15]. However, to our knowledge, only a small number of studies have investigated the effect of caudal block in the laparoscopic surgery for upper abdomen [14, 16], and few of them have investigated the impact of caudal block on opioid sparing during pediatric surgeries. Therefore, we conducted a prospective study to investigate the effect of caudal block on upper abdominal surgery under laparoscopy. We hypothesized that caudal block is able to reduce perioperative fentanyl use and produce good postoperative analgesic effects when compared to no caudal block. The primary outcome variable is perioperative fentanyl use.

## Methods

The study was approved by the China Medical University’s Institutional Review Board and written informed consent was obtained from all subjects participating in the trial. After receiving written consent from each patient’s guardian, we enrolled 96 pediatric patients (aged 6 months to 8 years, American Society of Anesthesiologists (ASA) grade I or II) who were scheduled to undergo elective laparoscopic upper urinary tract surgery. The upper urinary tract surgery includes pyeloplasty, nephrectomy, heminephrectomy, ureteroureterostomy, pyeloureterostomy and ureteral reimplantation. Pediatric patients who were allergic to local anesthetics and not suitable for caudal block (such as malformation of spine, infection around puncture site, and coagulation dysfunction, etc.) were excluded from this study. This study was conducted from May, 2018 to October, 2018 and adhered to CONSORT guidelines.

A total of 96 patients were equally randomized to a non-block group (no caudal block were performed), a ROP1.0 group (patients received caudal block with 1 mL/kg of 0.15% ropivacaine and 1 μg/kg fentanyl), and anROP1.3 group (patients received caudal block with 1.3 mL/kg of 0.15% ropivacaine and 1 μg/kg fentanyl) by stratified blocked randomization. Each group had the same number of patients (*n* = 32). Information regarding the patient’s age, gender, body weight, ASA grade, diagnosis and types of surgery was collected. All patients were from Shengjing hospital of China Medical University.

After the patients were transferred to the operating room, standard monitoring such as non-invasive blood pressure (NIBP), heart rate (HR), electrocardiography and pulse oximetry including pulse oxygen saturation (SpO_2_) was performed throughout the procedure. All patients received the standard general anesthesia induction procedure: fentanyl 1μg/kg, etomidate 0.3 mg/kg, succinylcholine 1.5 mg/kg and endotracheal intubation. Cisatracurium 0.05 mg/kg was used to provide continuous muscle relaxation. Sevoflurane of 1.0–1.3 minimal alveolar concentration (MAC) with 50% nitrous oxide was used to maintain anesthesia during the surgery. The tide volume was set to 6–10 mL/kg to guarantee that the end-tidal CO_2_ (ETCO_2_) was between 35 and 45 mmHg.

After endotracheal intubation, all patients were put in the left lateral position. Patients in the ROP1.0 group received caudal block with 1 mL/kg of 0.15% ropivacaine and 1 μg/kg fentanyl, while patients in the ROP1.3 group received caudal block with 1.3 mL/kg of 0.15% ropivacaine and 1 μg/kg fentanyl. The needle used in caudal block was 23G, and the injection speed of ropivacaine in the ROP1.0 and the ROP1.3 group was 0.5 mL/second. All the injections were performed with the guidance of ultrasound and by the same anesthesiologist. After caudal block, a sterile sticker was attached to the puncture site. In order to keep the observer blind to the allocating information, patients in the non-block group also had a same sterile sticker at the same site.

After the procedure of caudal block, the anesthesiologist who performed caudal block left the operating room, another anesthesiologist, who was blind to the grouping information, enter the operating room and managed the patients intraoperatively. Also he began to record the frequency of hemodynamic fluctuation. Hemodynamic fluctuation was defined as NIBP and (or) HR increasing or decreasing by more than 30% of the baseline and lasting for more than 2 min. If HR decreased by more than 30% of the baseline, atropine 0.01 mg/kg was given intravenously. If NIBP or HR increased by more than 30% of the baseline, an extra of 0.5 μg/kg fentanyl was given intravenously according to the anesthesiologist’s experience in hemodynamics fluctuation. The total amount of fentanyl used during surgery and the times of hemodynamic fluctuation were also recorded.

The site of laparoscopic port was decided by the surgeon for a successful procedure. Usually there needs to be three ports, one very close to the umbilicus (T10 dermatome), one at the intersection between arcus costarum and midclavicular line (T8 dermatome), and the last one near the iliac fossa (T12 dermatome). The pressure for laparoscopy was 8 mmHg, and the flow of carbon dioxide was 8 L/min.

Thirty minutes before the end of the surgery, patients were intravenously given ondansetron 100 μg/kg (to a maximum of 4 mg) and ketorolac 0.5 mg/kg (to a maximum of 15 mg). At the end of the surgery, patients received infiltrated anesthesia of ropivacaine around the laparoscopic port. After waking up naturally, patients were extubated when the extubation criteria were met and were transferred to the Post Anesthesia Care Unit (PACU).

In PACU, pain evaluation was performed by using the FLACC (Face Legs Activity Cry Consolability) score described by previous studies [15, 17, 18]. The same observer, who was blind to the allocating information, investigated the FLACC score. When the FLACC score was higher than 4, a rescue dosage of 0.5 μg/kg fentanyl was given to the patient intravenously. The FLACC score was re-investigated after 15 min of rescue fentanyl injection. If it was still higher than 4, another dosage of 0.5 μg/kg fentanyl was given. The FLACC score, the number of patients needing rescue fentanyl, the total amount of rescue fentanyl, side effects such as nausea and vomiting, and the time of PACU stay were also investigated.

After the PACU stay, patients were transferred to the ward, and the FLACC score of every 8 h in ward was also investigated. If the patients suffered from acute pain, oral paracetamol 15 mg/kg was prescribed. The number of patients needing NSAIDs, time to the first NSAIDs use in ward and side effects were also evaluated.

### Statistical analysis

Continuous variables such as fentanyl usage, the FLACC score and the time of PACU stay were presented as mean ± standard deviation (SD) or median with interquartile range. Categorical variables such as the frequency of hemodynamic fluctuation and the number of patients needing rescue fentanyl were presented as absolute numbers. Before comparing the differences in continuous variables, D’Agostino-Pearson omnibus normality test was performed to investigate data distribution. Since fentanyl usage, the FLACC score and the time of PACU stay were normally distributed among groups, one-way ANOVA and Bonferroni correction were used one after another to investigate the difference between groups. Differences in categorical variables were analyzed by a Chi-square test or a Fisher’s exact test. For all statistical analysis, a *P* value of less than 0.05 was considered statistically significant.

In order to calculate the sample size needed for this study, a pilot study was performed. The pilot data showed that the perioperative fentanyl use (the primary outcome variable of this study) of the non-block group, the ROP1.0 group and the ROP 1.3 group were1.87 ± 0.35 μg/kg, 1.57 ± 0.32 μg/kg and 1.29 ± 0.29 μg/kg respectively. Since we assumed that the type I error was 0.05 and the power was 0.9, the sample size calculated based on the non-block group and the ROP1.0 group was 24, while the sample size calculated based on the ROP1.3 group and the ROP1.0 group was 27. Considering the 10% dropout rate, we increased the sample size by 20% and the final result was 32 per group.

## Results

A total of 96 pediatric patients were enrolled in this study, 32 in each group. Four patients withdrew from the non-block group because of the change of surgery method and incomplete data. Two patients in the ROP1.0 group and two patients in the ROP1.3 group were left out because of incomplete data and refusal of participation. The final numbers of patients in the non-block group, the ROP1.0 group and the ROP 1.3 group were 28, 30 and 30 respectively. There was no significant difference between these three groups regarding age, body weight, gender, ASA grade and operating time (Table 1).

**Table 1:** Demographic data of patient

	Non-block(*n* = 28)	ROP1.0(*n* = 30)	ROP1.3(*n* = 30)
Gender, n (%)			
Female	15 (54)	14 (48)	17 (57)
Male	13 (46)	16 (52)	13 (43)
Age, years; median (min-max)	2.4 (0.5–6.5)	2.8 (0.5–7.4)	2.6 (0.5–7.5)
weight, Kg; median (min-max)	13.4 (6.8–22.6)	13.1 (8.2–21.5)	12.6 (7.8–23.4)
ASA I/II	20/8	21/9	18/12
Operating time, mins; median (min-max)	134 (95–245)	155 (100–264)	143 (102–255)

Values are presented by median (min-max) or numbers of patients

Non-block: patients received no caudal block

ROP1.0: patients received caudal block with 1.0 mL/kg ropivacaine

ROP1.3: patients received caudal block with 1.3 mL/kg ropivacaine

**Fig. 1:**
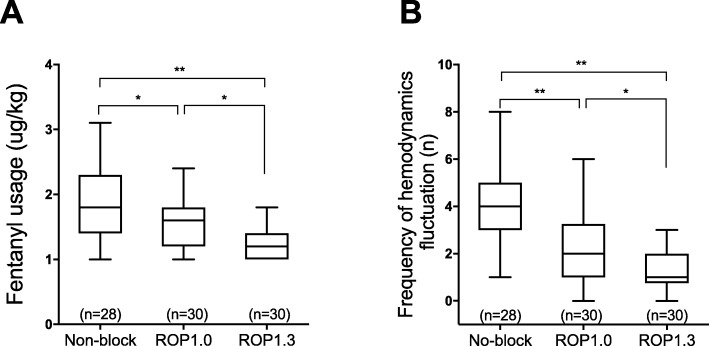
Comparisons of fentanyl usage and frequency of hemodynamics fluctuation during operation between groups. **a** Comparisons of intraoperative fentanyl usage. **b** Comparisons of intraoperative frequency of hemodynamics fluctuation. The whisker of boxplot stands for the min and max, line in box stands for median. * *P* < 0.05, ** *P* < 0.01. Non-block: patients received no caudal block, ROP1.0: patients received 1.0 mL/kg of 0.15% ropivacaine, ROP1.3: patients received 1.3 mL/kg of 0.15% ropivacaine

First we investigated the perioperative fentanyl usage (fentanyl use in the operation and in PACU), which is the primary outcome of this study. As depicted in Fig. 1a, caudal block significantly decreased perioperative fentanyl usage (ROP1.0 vs. non-block: 1.57 ± 0.38 μg/kg vs. 1.85 ± 0.56 μg/kg, *P* = 0.027; ROP1.3 vs. non-block: 1.27 ± 0.24 μg/kg vs. 1.85 ± 0.56 μg/kg, *P* < 0.001). Compared to the ROP1.0 group, patients in ROP1.3 group needed less fentanyl (ROP1.0 vs. ROP1.3: 1.57 ± 0.38 μg/kg vs. 1.27 ± 0.24 μg/kg, *P* = 0.014). The frequency of hemodynamic fluctuation during operation was also recorded. As shown in Fig. 1b, caudal block maintained more stable hemodynamics when compared with patients with no caudal block (ROP1.0 vs. non-block: 2.27 ± 1.57 times vs. 4.14 ± 1.75 times, P < 0.001; and ROP1.3 vs. non-block: 1.3 ± 0.99 times vs. 4.14 ± 1.75 times, *P* < 0.001), and patients in the ROP1.3 group showed more stable hemodynamics than patients in the ROP1.0 group (ROP1.3 vs. ROP1.0: 1.33 ± 0.99 times vs. 2.27 ± 1.57 times, *P* = 0.043).

**Fig. 2:**
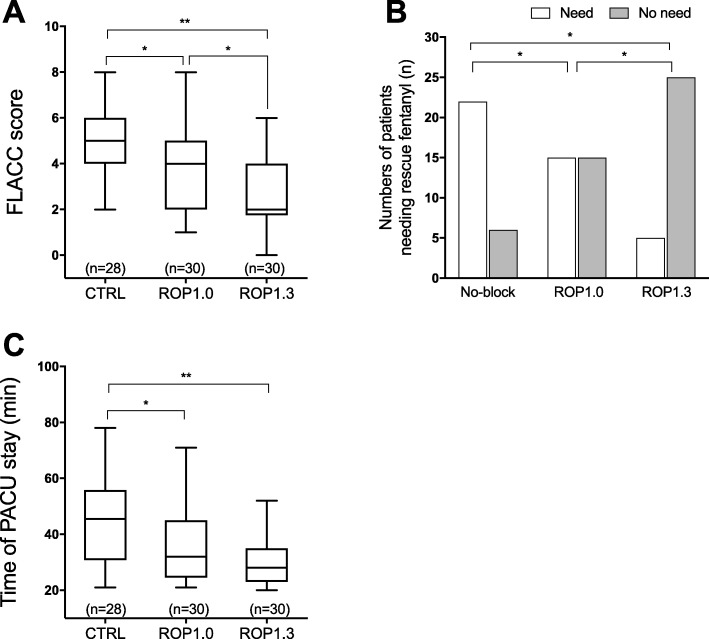
Comparisons of FLACC score, numbers of patients needing rescue fentanyl and time of stay in PACU between groups. **a** Comparison of FLACC score in PACU. **b** Comparison of numbers of patients needing rescue fentanyl in PACU. **c** Comparison of time of PACU stay. The whisker of boxplot stands for the min and max, line in box stands for median. * *P* < 0.05, ** *P* < 0.01. Non-block: patients received no caudal block, ROP1.0: patients received 1.0 mL/kg of 0.15% ropivacaine, ROP1.3: patients received 1.3 mL/kg of 0.15% ropivacaine

In the PACU, the FLACC score, the number of patients needing rescue fentanyl and PACU stay were also recorded. As shown in Fig. 2a, patients who received caudal block exhibited a significantly lower FLACC score (ROP1.0 vs. non-block: 3.87 ± 1.76 vs. 5.00 ± 1.67, *P* = 0.032; and ROP1.3 vs. non-block: 2.70 ± 1.64 vs. 5.00 ± 1.67 times, *P* < 0.001). In accordance with the FLACC score, more patients in the non-block group needed rescue fentanyl in the PACU when compared with patients in the ROP1.0 and the ROP1.3 groups (Fig. 2b). The average time of PACU stay of the non-block group was 44.29 ± 15.23 min, significantly longer than that of the ROP1.0 and the ROP1.3 groups (Fig. 2c).

**Fig. 3:**
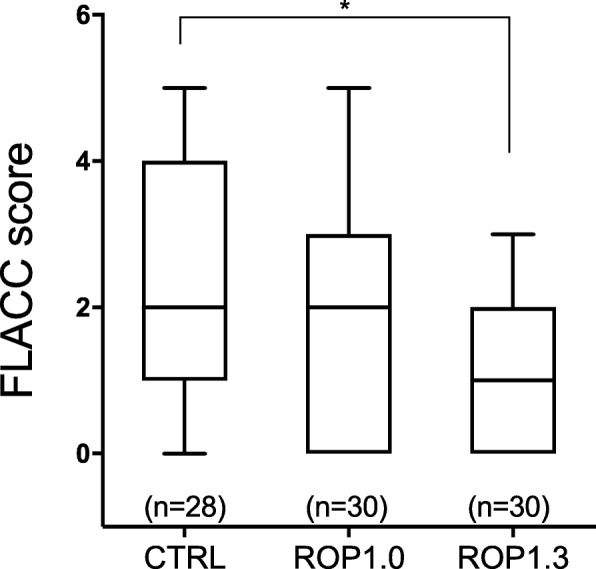
Comparison of FLACC score of 8 h after operation The whisker of boxplot stands for the min and max, line in box stands for median. * *P* < 0.05, Non-block: patients received no caudal block, ROP1.0: patients received 1.0 mL/kg of 0.15% ropivacaine, ROP1.3: patients received 1.3 mL/kg of 0.15% ropivacaine

Postoperative data such as the FLACC score 8 h after surgery, number of patients needing NSAIDs, time to first NSAIDs use in ward and side effects were also evaluated. As shown in Fig. 3, the postoperative FLACC score of the ROP1.3 group was significantly lower than that of the non-block group (ROP1.3 vs. non-block: 1.07 ± 1.01 vs. 2.45 ± 1.35, *P* = 0.003). Patients with no caudal block needed NSAIDs earlier, and more patients needed postoperative NSAIDs when compared with patients in the ROP1.0 and the ROP 1.3 groups (Table 2). There were no significant differences in side effects between the groups. In the non-block group, 6 patients had postoperative vomiting, while the number was 3 and 2 in the ROP1.0 and the ROP 1.3 groups. Only 1 patient had postoperative motor weakness in the ROP1.3 group, but the patient recovered after 24 h. No patients in these groups exhibited bleeding or infection at the puncture site.

**Table. 2:** Postoperative data and side effects

	n-block(*n* = 28)	ROP1.0(*n* = 30)	ROP1.3(*n* = 30)
number of patients needing NSAIDs (n, %)	28 (100%)	17 (57%)*	9 (30%)**#
first NSAIDs time (min)	125 (62–242)	562 (362–845)**	675 (453–987)**
Side effects			
Vomiting (n, %)	6	3	2
Motor weakness (n, %)	0	0	1
Bleeding or infection of puncture site (n, %)	0	0	0

Data were presented as number and ratio or median (min-max)

* *P* < 0.05, ** *P* < 0.01 Compared to the Non-block group

# *P* < 0.05, compared to the ROP1.0 group

## Discussion

To the best of our knowledge, our study was the first prospective study to investigate the effect of caudal block on pediatric upper urinary tract surgery under laparoscopy. We found that caudal block with 1.3 mL/kg of 0.15% ropivacaine reduced perioperative fentanyl use and maintained stable hemodynamics during laparoscopic surgery in comparison with the non-caudal block group and the ROP1.0 group. Moreover, patients in the ROP1.3 group showed lower FLACC score, delayed NSAIDs need, low occurrence of vomiting and reduced rescue fentanyl need.

Opioids have been used to control perioperative pain for many years, but excessive use of opioids often leads to a series of side effects, such as postoperative nausea and vomiting, respiratory depression, drowsiness that hampers recovery, prolonged hospital stay and increased medical costs [8, 9, 19]. Nowadays, a variety of techniques, such as incision infiltrated with local anesthetics, transverse abdominis plane (TAP) block, caudal block and the combined usage of NSAIDs, are performed to reduce the use of opioids and its related side effects [20–22]. In this study, we found that caudal block with 1.3 mL/kg of 0.15% ropivacaine significantly reduced the use of fentanyl and the occurrence of vomiting. It also shortened the time of PACU stay.

There were several studies investigating the effect of local anesthesia on pediatric surgeries [12, 23, 24]. Sandeman et al. investigated the effect of TAP on laparoscopic appendicectomy performed on children and found that TAP did not have a positive effect on postoperative analgesia when compared to local anesthetic port-site infiltration [23]. Nitin et al. [15] compared the effect of TAP and caudal block on children undergoing lower abdominal surgery and found that children who received caudal block suffered from higher occurrence of pain after surgery than children who received TAP, while caudal block had longer postoperative analgesia duration than TAP. In their study, the amount of local anesthetics was 0.75 mL/kg of 0.25% bupivacaine, lower than the amount used in our study (1.3 mL/kg). Faasse et al. [14] investigated the effects of TAP and caudal block on children in urologic robot-assisted laparoscopic surgery and found that patients with caudal block needed less opioids during surgery and less postoperative antiemetics. In our study, caudal block with 1.3 mL/kg of 0.15% ropivacaine decreased perioperative fentanyl use and the occurrence of postoperative vomiting, a fact that was consistent with the previous study. However, the study carried out by Faasse was a retrospective one with a moderate sample size, and the kind and amount of local anesthetics used were not the same. In addition, in our study the ROP1.3 group also presented reduced pain scores, smaller number of patients needing post-operative NSAIDs and late first post-operative NSAIDs needed when compared with the non-block group, which was not the same as the Faasse’s study.

Numerous studies have investigated the relationship between the amount and injection speed of local anesthetics and the level of cranial spread [25–27]. Brenner et al. [28] used ultrasound to assess the cranial spread of different volumes of local anesthetics during caudal block performed on children. They found that the maximal level of cranial spread was T10, and there was no statistical difference between 1.0 mL/kg ropivacaine and 1.3 mL/kg ropivacaine. Triffterer et al. [29] tested the cranial spread of local anesthetics at different injection speeds and found that the injection speed did not affect cranial spread during caudal block performed on pediatric patients. The maximal level of cranial spread detected by ultrasound was L1 when using 1.0 mL/kg of ropivacaine. Lundblad et al. [30] used ultrasound to investigate the segmental distribution of high volume caudal anesthesia and found that the maximal cranial spread level was T9 when using 1.5 mL/kg of 0.2% ropivacaine. In our study, when the surgeon performed the incision at the T8 level, a large part of patients in the ROP1.3 group showed stable hemodynamics. Several reasons may account for this phenomenon. First, in our study, we incorporated the use of fentanyl and maintained the MAC between 1.0 and 1.3, which may result in a stable hemodynamics. Second, in our study, all of the surgeries were performed under laparoscopy, and the increased abdominal pressure caused by pneumoperitoneum may affect the spread of local anesthetics. Further study needs to be done to investigate the relationship between pneumoperitoneum and local anesthetics spread.

### Limitation

1. In our study, no placebo injection was performed in the non-block group (control group). We simply attached a sterile sticker to cover the same site of caudal block groups. We believed that a placebo injection was an invasive injection that could be harmful to the patient. 2. In previous studies, the maximal level of cranial spread was about T10, while in this study, when the surgeon performed an incision at the T8 level, most of the patients in the ROP1.3 group showed stable hemodynamics. That said, we cannot directly conclude that the maximal level of cranial spread was T8, and further investigation needs to be done to confirm that. 3. Previous studies showed that the maximal level of cranial spread in elder patients was lower than that in younger patients, while in our study we did not observe a significant difference between younger and older patients due to the small sample size. Further study with a larger sample size needs to be done to investigate the relationship between age and caudal block effect.

## Conclusion

Caudal block with 1.3 mL/kg of 0.15% ropivacaine reduced fentanyl use during laparoscopic upper urinary tract surgery on pediatric patients and produced good postoperative analgesia when compared with no caudal block and caudal block with 1.0 mL/kg of 0.15% ropivacaine usage.

## Data Availability

The datasets analyzed during the current study are available from the corresponding author on reasonable request.

## Abbreviations

ASA: American Society of Anesthesiologists
ETCO_2_: End-tidal CO_2_
FLACC score: Face Legs Activity Cry Consolability score
HR: Heart rate
MAC: Minimal alveolar concentration
NIBP: Non-invasive blood pressure
NSAIDs: Non-steroidal antiinflammatory drugs
PACU: Post anesthesia care unit
ROP: Ropivacaine
SpO2: Pulse oxygen saturation
